# Metagenomics of the Water Column in the Pristine Upper Course of the Amazon River

**DOI:** 10.1371/journal.pone.0023785

**Published:** 2011-08-19

**Authors:** Rohit Ghai, Francisco Rodriguez-Valera, Katherine D. McMahon, Danyelle Toyama, Raquel Rinke, Tereza Cristina Souza de Oliveira, José Wagner Garcia, Fernando Pellon de Miranda, Flavio Henrique-Silva

**Affiliations:** 1 Evolutionary Genomics Group, Departamento de Producción Vegetal y Microbiologia, Universidad Miguel Hernández, San Juan de Alicante, Alicante, Spain; 2 Laboratory of Molecular Biology, Departamento de Genética e Evolução, Universidade Federal de São Carlos, São Carlos, SP, Brasil; 3 Universidade Federal do Amazonas, Setor Sul do Campus Universitário, Manaus, AM, Brasil; 4 Noosfera Projetos Especiais Ltda, São Paulo, SP, Brasil; 5 Petróleo Brasileiro S.A. – Petrobras, Centro de Pesquisas e Desenvolvimento Leopoldo Américo Miguez de Melo, Rio de Janeiro, RJ, Brasil; Université Paris Sud, France

## Abstract

River water is a small percentage of the total freshwater on Earth but represents an essential resource for mankind. Microbes in rivers perform essential ecosystem roles including the mineralization of significant quantities of organic matter originating from terrestrial habitats. The Amazon river in particular is famous for its size and importance in the mobilization of both water and carbon out of its enormous basin. Here we present the first metagenomic study on the microbiota of this river. It presents many features in common with the other freshwater metagenome available (Lake Gatun in Panama) and much less similarity with marine samples. Among the microbial taxa found, the cosmopolitan freshwater acI lineage of the actinobacteria was clearly dominant. Group I Crenarchaea and the freshwater sister group of the marine SAR11 clade, LD12, were found alongside more exclusive and well known freshwater taxa such as *Polynucleobacter*. A metabolism-centric analysis revealed a disproportionate representation of pathways involved in heterotrophic carbon processing, as compared to those found in marine samples. In particular, these river microbes appear to be specialized in taking up and mineralizing allochthonous carbon derived from plant material.

## Introduction

River water, around which civilizations flourish, is only a very small percentage (0.006%) of the total freshwater on earth, and a miniscule 0.0002% of the total water in the hydrosphere [1]. Even so, freshwater habitats like rivers, streams, lakes and wetlands, provide invaluable ecosystem services to human populations in the form of drinking water, recreation, and fisheries. They also play a previously underestimated but surprisingly important role in the oxidation, storage, and release of terrestrial carbon, thereby affecting global carbon budgets [2], [3], [4]. The Amazon river basin is the largest river basin in the world, comprising ∼40% of the total area of the continent of South America. The Amazon river itself is by far the largest river in the world in terms of volumetric discharge (6.3 trillion m^3^/year), with a length of 6280 km, a catchment area the size of ∼7000 square kilometres, and runoff amounting to nearly 15% of the total runoff of all the rivers in the world together. The tropical rainforest surrounding the river is an extraordinarily diverse ecosystem, boasting thousands of plant and animal species, many endemic to it, with several regions still untouched by anthropogenic pressure. However, such a situation may not continue for long since the extent of pristine waters in these regions might be dramatically reduced in the future. This, of course, has serious consequences for the macrofauna and flora, the most visible components of the ecosystem. However, the less visible, microscopic component of this habitat has been largely ignored. We have practically no information on the major microbial species that dominate the Amazon River.

Freshwater habitats harbor microbial taxa distinct from those routinely detected in marine and terrestrial ecosystems [5], [6]. Notably, surveys targeting 16S rRNA genes in freshwater lakes and reservoirs (reviewed in [7]) have identified several “cosmopolitan” and prevalent freshwater lineages of Betaproteobacteria [8], [9], [10], [11], [12] and Actinobacteria [13], [14], [15], [16], [17]. Other commonly recovered lineages belong to the Bacteroidetes, Verrucomicrobia, and Alphaproteobacteria. Cyanobacteria and Gammaproteobacteria are retrieved frequently in particular types of lakes (e.g. highly productive or polluted). Much has been learnt about the primary factors determining lake bacterioplankton community composition, such as trophic status [18], [19], pH [20], [21], landscape position [22], and retention time [20]. Comparatively fewer studies have been conducted on lotic systems, and these frequently focused on rivers or streams much smaller than the Amazon. Many have specifically targeted biofilm communities [23]. Pelagic or suspended bacterioplankton in large flowing rivers tends to comprise freshwater taxa routinely detected in lakes [24], [25], [26], but very little is known about their traits or functions. Though there have been instances of 16 s rRNA based surveys of river waters [27], surprisingly, only three metagenomic studies exploring the functional capabilities of freshwater microbes have been published to date, representing tropical lakes Gatun (Panama) [28], and Samsonvale (Australia) [29] and the temperate eutrophic Lac du Bourget (France) [30]. Apart from the large sequencing effort for the Lake Gatun sample, the other studies were relatively small (0.15 Mb Samsonvale, 11 Mb Lac du Bourget).

This is the first study to present the analysis of a large (375 Mb) freshwater metagenomic dataset. Besides, we provide a first glimpse into the functional characteristics of microbiota of the water column of the Solimões-Amazon (henceforth referred to as the Amazon) river in its upper reaches (about 420 km upstream from Manaus), a region relatively pristine with little (if any) human impact and more than 1300 km from the nearest ocean (the Atlantic). Total DNA from a single sample from the mid water column, which provides us with a snapshot of this part of the river, was directly 454 pyrosequenced providing 375 Mb of sequence data. Here we present the analysis of this dataset.

## Results and Discussion

### Sample Description

The source of the Amazon river is in the Andes mountains. Several rivers gradually merge together (mainly the Rio Ucayali and Rio Marañón from the Peruvian Andes) to form the Amazon proper. The river and its tributaries, including the floodplains, cover about 300,000 km^2^. The floodplains are of fundamental importance to the flora and fauna of the region. Three types of streams are usually distinguished in the Amazon hydrological basin; clearwater, white-water and blackwater [31]. Clearwater streams are low in suspended sediment and dissolved carbon, whereas white-water streams have high sediment concentrations. Both types have a near-neutral pH. Blackwater streams, on the other hand, are low in suspended sediment but are rich in dissolved organic matter and have a lower pH (about 4–5). The sample analyzed here is a typical example of mainstem white water. The sample was collected at a site nearly 400 km upstream from Manaus, Brazil, at the end of the dry season (September, average rainfall for the month 50 mm) and was taken from mid stream and mid waters (8 m depth with the bottom at 15 m) (Figure S1). This site is upstream from the Coari Terminal of Petrobras (Brazil's state-controlled petroleum company), and still in a pristine condition. Physicochemical parameters of the sample are provided in Table S1. One full plate of 454 sequencing yielded nearly one million reads (n = 1153502), with an average read length of 325 bases. This amounts to a total of 375 Mb, the largest reported so far for any freshwater habitat. The next largest dataset from a freshwater metagenome is that of Lake Gatun, a freshwater lake near the Panama Canal that was sampled during the Global Ocean Survey (GOS) [28], and the only freshwater sample in that collection (total 315 Mb). We note, however, that the Lake Gatun sample comprised the 0.1–0.8 µm size fraction from the near surface (2 m deep) while our Amazon sample constituted the 0.2–5 µm fraction and was taken from mid depth (8 m).

### Comparative characteristics of freshwater metagenomic datasets

**Figure 1:**
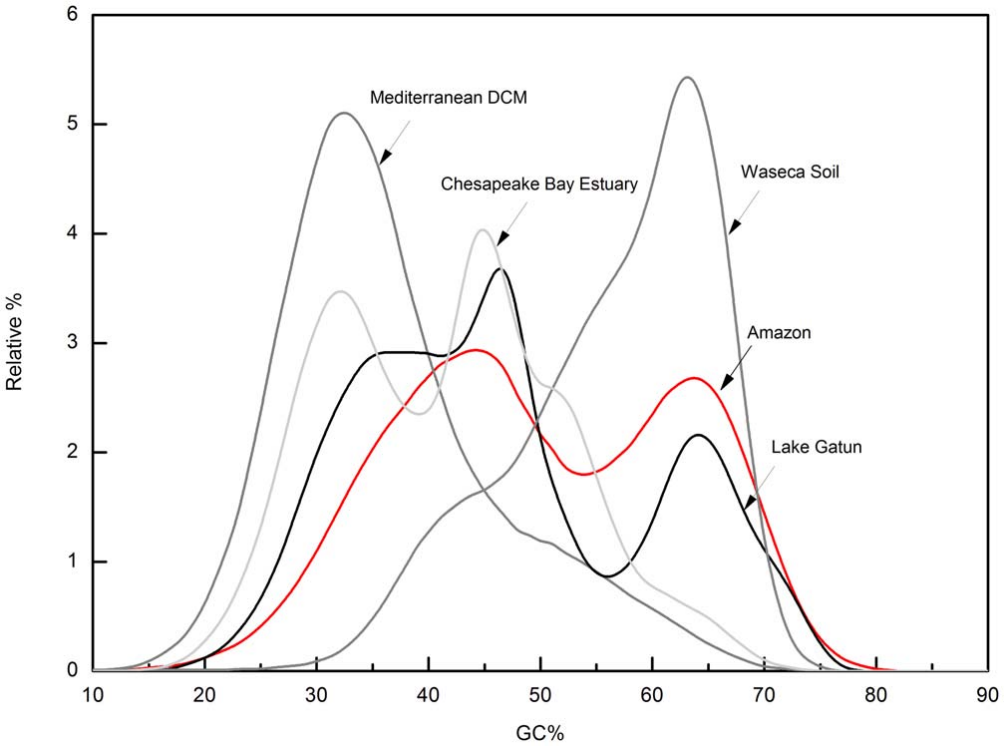
GC content of diverse metagenomic datasets. The GC% profile of the Amazon reads is shown (red) in comparison to a freshwater datasets (Lake Gatun), an estuary (Chesapeake Bay), a typical marine sample (Mediterranean Deep Chlorophyll Maximum) and a soil metagenome (Waseca County Soil).

The GC content plot of metagenomic datasets is often characteristic with widely different values and shape depending on the habitat. Two extremes are marine oligotrophic water samples with a marked low GC peak (∼32%) and soil with a distinctly higher GC peak (∼65%) (Figure 1). The GC% of the Amazon dataset, along with Gatun had a distinctive bimodal distribution with almost equally sized peaks of low and high GC% (∼45% and 65%) (Figure 1). The low GC peak of ca. 45% might be a freshwater hallmark since it was found in both freshwater metagenomes, but its difficult to make a generalization based on only two samples. However, in both estuary samples from the GOS datasets two peaks were again observed, one coinciding with the purely marine waters (∼35%) and the other (∼45%) with the low GC% peak of the Amazon and Gatun datasets.

**Figure 2:**
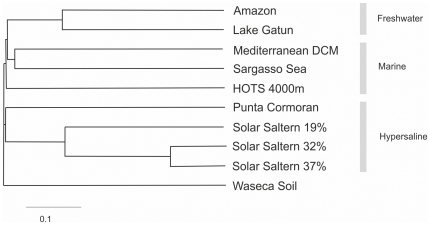
Metagenomic dataset similarity to each other. The tree shows the relatedness of diverse metagenomic datasets based on Jaccard distance derived from all versus all comparisons of reads with BLASTN. Datasets of different salinities were chosen for the comparison. Freshwater: Amazon, Lake Gatun; Marine: Mediterranean DCM, Sargasso Sea GS000a, HOTS 4000 m; Hypersaline: Punta Cormoran 6% (salinity) and Santa Pola salterns of three different salinities 19%, 32% and 37%). Waseca county soil dataset was used as an outgroup to construct the tree (see methods).

Salinity has been shown to be a critical environmental factor in determining community structure of the microbiota [32]. We compared the sequences retrieved here with other aquatic metagenomic datasets with different salinities using all versus all comparisons of reads with BLASTN. The most similar sample to the Amazon metagenome was the freshwater sample from Lake Gatun, followed by two estuary samples (Figure 2 and Figure S2). As a reference we included in the comparison metagenomes from hypersaline ponds and the divergence between the two freshwater datasets was similar to that found between a 19% (5 times seawater salinity) and 37% (10 times seawater salinity). We know that these two environments still share many microbes at least at the level of genus [33]. A closer comparison to the Lake Gatun dataset revealed that the Amazon reads could overlap with nearly 55% of the Lake Gatun sequences (using Amazon metagenome reads as queries, 50% query coverage and with %identity of >90%), indicating a high amount of similar sequences in these two habitats, i.e. extremely closely related microbes inhabiting both locations. Reference genomes were used to recruit reads common to both datasets, to determine which organisms might be common to both systems (Table S2).

### Microbiota of the Amazon

**Figure 3:**
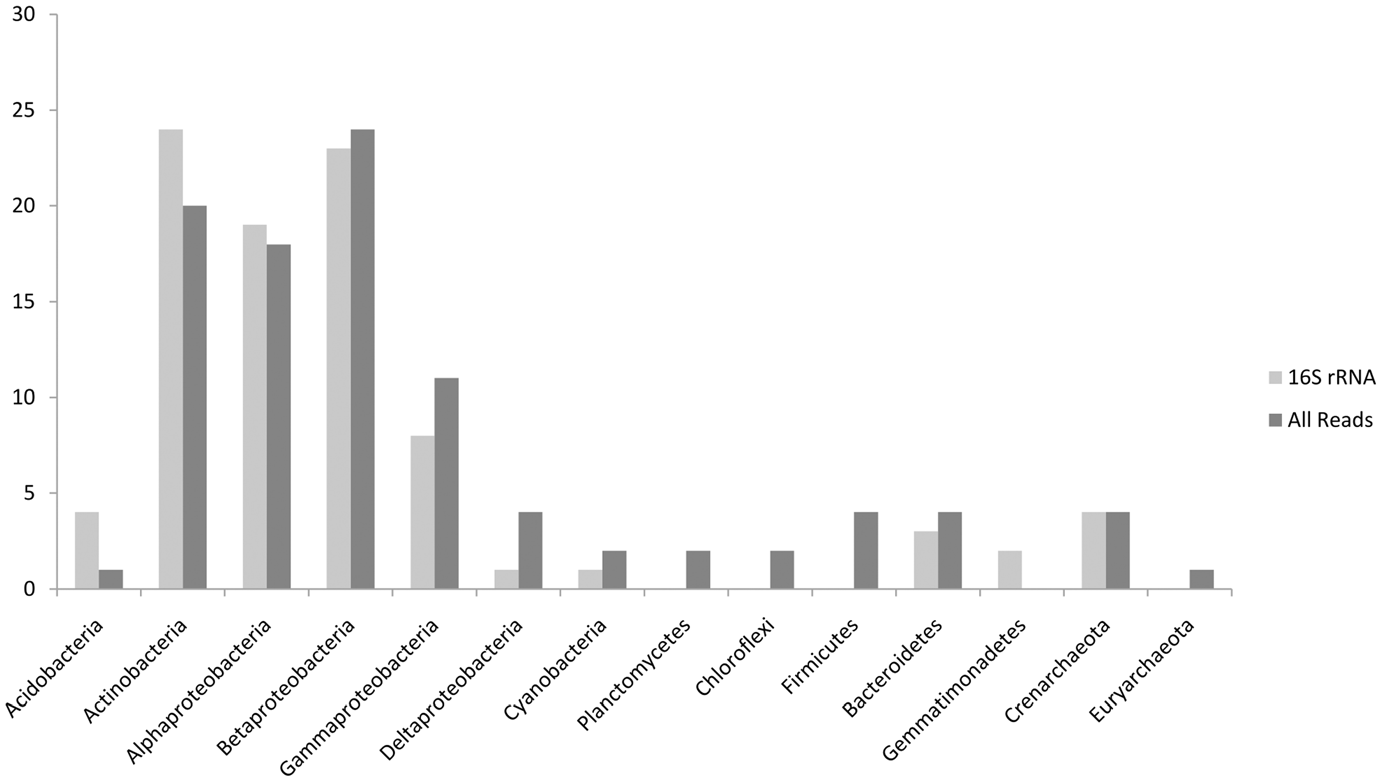
Phylogenetic profile of the Amazon metagenome. Phylogenetic profile of the Amazon metagenome is shown using two different approaches, one, using the 16 s rRNA sequences gathered from the metagenome, and the other using all the reads and comparing against sequenced microbial genomes (using the MG-RAST server).

To analyze the community structure of the mainstem Amazon we have used the results from the direct 454 pyrosequencing and classified the 16S rRNA gene fragments recovered from the metagenome (722 sequences) (Figure 3). The advantage of this approach is that it is free from any amplification (PCR) or cloning bias [34]. The most frequently recovered 16S rRNA gene sequences were affiliated with the Actinobacteria and Proteobacteria (Alpha, Beta and Gamma) (Figure 3). We also attempted to bin all 1.1 million pyrosequencing reads with the MG-RAST server and found very similar results (∼49% % of reads could be assigned to a taxon) (Figure 3 and Table 1). Finally, the recruitment of genomes of representative strains that were detected was analyzed to assess the coverage and the overall similarity of the relatives in the Amazon dataset (Figure S3).

**Table 1:** Sequenced genomes with most hits to the Amazon metagenome (using the MG-RAST server).

Taxonomic Group	Organism Name	# Hits
Crenarchaeota	*Nitrosopumilus maritimus* SCM1	17012
Betaproteobacteria	*Polynucleobacter* sp. QLW-P1DMWA-1	16460
Gammaproteobacteria	*Acinetobacter baumannii* ATCC 17978	13424
Alphaproteobacteria, SAR11 cluster	*Candidatus* Pelagibacter ubique HTCC1062	12269
*Actinobacteria*	*Acidothermus cellulolyticus* 11B	10656
Betaproteobacteria	*Polaromonas* sp. JS666	10116
Acidobacteria	*Solibacter usitatus* Ellin6076	10059
Betaproteobacteria	*Methylobacillus flagellatus* KT	8980
*Actinobacteria*	*Streptomyces scabiei* str. 87.22	8741
*Actinobacteria*	*Janibacter* sp. HTCC2649	8697
*Actinobacteria*	*Streptomyces avermitilis* MA-4680	7909
*Actinobacteria*	*Thermobifida fusca* YX	7628
Betaproteobacteria	*Rubrivivax gelatinosus* PM1	7524
*Actinobacteria*	*Streptomyces coelicolor* A3(2)	7204
Betaproteobacteria	*Herminiimonas arsenicoxydans*	7046
*Actinobacteria*	*Kineococcus radiotolerans* SRS30216	6577
Betaproteobacteria	*Rhodoferax ferrireducens* DSM 15236	6524
*Actinobacteria*	*Frankia* sp. EAN1pec	6177
Betaproteobacteria	*Delftia acidovorans* SPH-1	5496
Betaproteobacteria	*Acidovorax avenae* subsp. citrulli AAC00-1	5495

**Figure 4:**
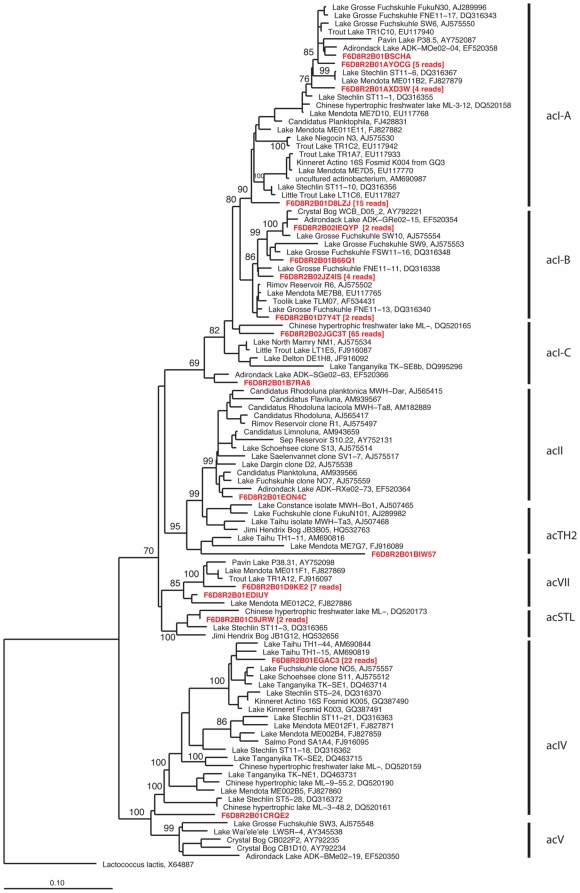
Phylogenetic affiliation of the Actinobacterial reads of the Amazon metagenome. Most of the 16S rRNA reads affiliated with the freshwater acI and acIV lineages. Phylogenetic reconstruction was conducted by maximum likelihood (RAxML) with near full length (>1300 nt) reference 16S rRNA gene sequences from a manually curated alignment [32] and highly variable positions masked. Metagenomic reads were added without altering tree topology using maximum parsimony criterion and a 50% base frequency filter in the ARB software package. Bootstrap values are indicated above nodes with greater than 60% support and the scale bar represents 10 base substitutions per 100 nt positions.

All approaches confirmed the presence of large numbers of Actinobacteria (20–25% of reads). Although this was not unexpected since they have been found in freshwaters of all kinds (still and flowing), [7], [13], [14], [17]. Actinobacterial genomes recruited smaller numbers individually but when pooled together they recruited the most (20% of binned reads). Actually, the available actinobacterial genomes recruited at a very low similarity (60% mean percentage identity in translated sequence comparisons) indicating a very distant relationship with the ones found in the Amazon. Cosmopolitan lineages of freshwater Actinobacteria have remained difficult to culture and thus no complete genomes are yet available. In the absence of complete genome information, we performed phylogenetic analysis of the actinobacterial rRNA reads in context of nearly full-length rRNA sequences from freshwater Actinobacteria recovered previously using PCR-based 16S rRNA gene cloning to determine more specifically which acintobacterial clades were present in the metagenome sample. A total of 135 reads were identified as containing partial 16S rRNA gene sequences related to Actinobacteria, and these were inserted into a maximum likelihood phylogenetic tree constructed with nearly full-length 16S rRNA sequences (Figure 4). Based on this analysis, most were affiliated with the acI lineage (73%) and with the acIV lineage (17%). A few were also assigned to the acII, acTH2, acVII, and acSTL lineages. Therefore, we infer that most of the metagenomic reads binned as Actinobacteria are derived either from acI or acIV, both of which are cosmopolitan freshwater taxa found in many different types of lakes, reservoirs, and rivers. It has been proposed that these have a photoheterotrophic lifestyle since rhodopsins affiliated to actinobacteria have been described (actinorhodopsins) in Lake Gatun [35]. In our dataset as well, the abundance of actinorhodopsins was evident (71 out of a total of 125).

**Figure 5:**
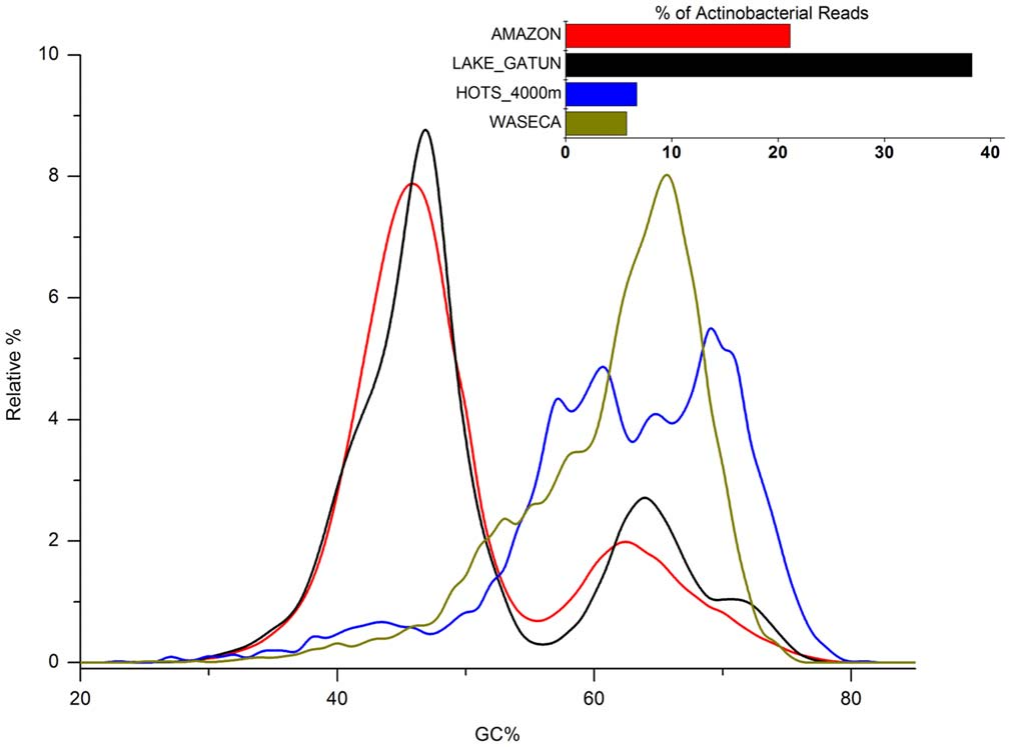
GC% of actinobacterial reads in diverse metagenomic datasets. The GC% of all reads assigned to actinobacteria in different datasets is shown. Datasets shown are the Amazon metagenome (red), Lake Gatun (black), HOTS 4000 m (deep blue), Waseca County Soil (Brown). The inset shows the % of actinobacterial reads in each dataset.

The individual reads adscribed to Actinobacteria were low GC (Figure 5), as has been recently described for the abundant freshwater actinobacteria [36]. It is interesting that the only large freshwater metagenome available in databases (Lake Gatun) also contained a massive fraction (close to 40%) of actinobacterial reads, explaining a large part of the sequence overlap between the two environments (∼47% of the overlap). A soil metagenome (Waseca County Soil Sample) and the deep sea HOTS 4000 m sample showed only the presence of high GC Actinobacteria. The other important taxa that contributed prominently to the low GC reads in the Amazon dataset were the Crenarchaea and Bacteroidetes (Figure S4).

Betaproteobacteria were the next most dominant group. They were represented by a diverse collection of organisms, with members of the *Polynucleobacter* (Pnec) and Methylophilaceae (betIV) lineages comprising 9% and 22% of betaproteobacterial rRNA reads, respectively. In addition, the *Polynucleobacter* sp. QLW-P1DMWA-1 genome recruited more reads than any other available bacterial genome, although at low similarity (Table 1 and Figure S3). The presence of *Polynucleobacter* relatives is not surprising as it is among the most well known and widely distributed bacterium that has been isolated from both lentic and lotic habitats all over the world [8], [11].

Even though archaea accounted for only about 6% of the 16S rRNA detected, the genome that recruited the most reads (3% of binned reads) was *Nitrosopumilus maritimus*, an ammonia oxidizing crenarchaeon isolated originally from a marine fish tank [37] (Table 1 and Figure S3). Crenarchaea were originally thought to be constrained to harsh environmental niches but several surveys have now demonstrated the ubiquity of crenarchaea in aquatic systems [38]. A few studies done in freshwater systems have demonstrated the role of group 1 crenarchaeota in ammonia-oxidation [39], [40]. We examined the abundance of archaeal reads in diverse metagenomic datasets and they always comprised a small percentage (less than 6%) of the total (Figure S5). For instance, the deep ocean HOTS 4000 m sample [41] which was the highest percentage of archaeal reads we found in the datasets we examined, contained 5.8% archaeal reads. Besides, only the Amazon and Lake Gatun datasets were found to contain more Crenarchaea than Euryarchaea, while the opposite was observed for a soil metagenome and the Mediterranean Deep Chlorophyll Maximum, and a selected sample from Sargasso Sea and the deep ocean HOTS 4000 m sample had nearly equal amounts of both taxa (Figure S5). It is clear that crenarchaea are numerically dominant in freshwater systems than in marine and thus probably have a greater role to play in nutrient cycling in both lentic and lotic habitats, as some previous PCR studies of 16S rRNA genes already indicated [42], [43], [44].

**Figure 6:**
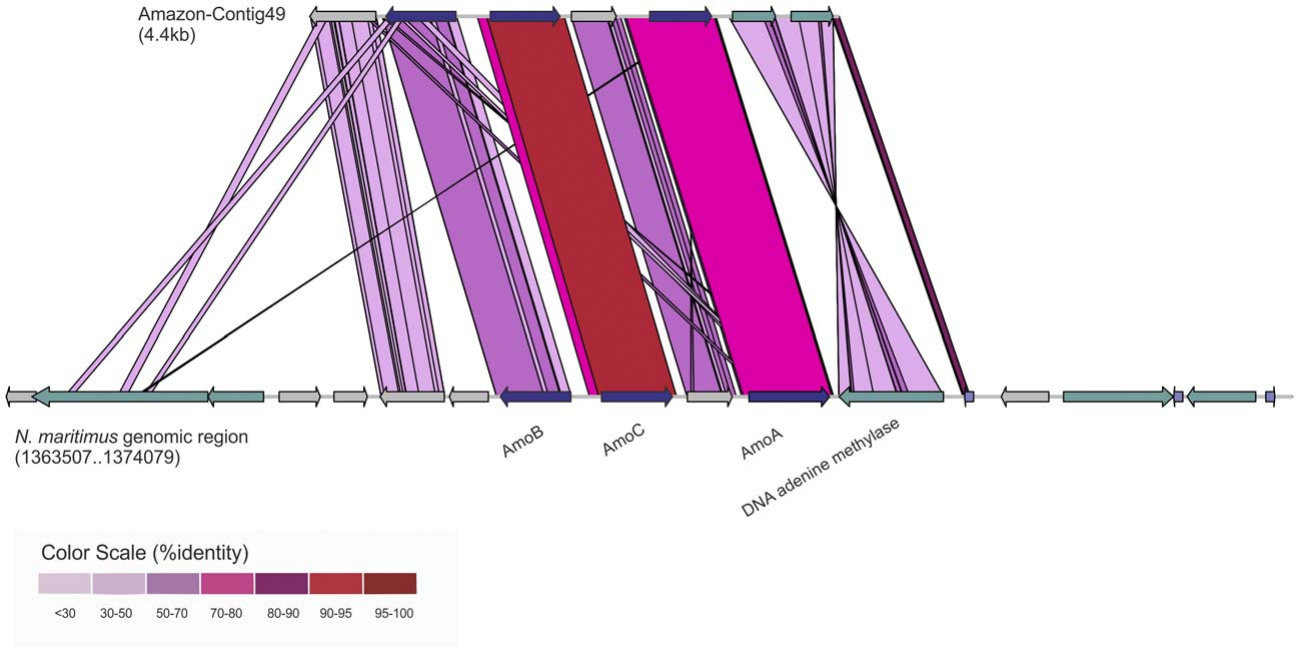
Synteny of assembled crenarchaeal contig from the Amazon metagenome to the *Nitrosopumilus maritimus* genomic region containing the genes for ammonia oxidation. The Amazon contig is shown on top and the *N. maritimus* genomic region is shown below. The colors indicate the level of %identity (using TBLASTX) between the sequences.

Assembly of the entire dataset using stringent cutoffs to avoid chimeric assemblies (see methods) yielded a total of 65 contigs larger than 3 kb. Of these, 16 were clearly crenarchaeal and 7 were of alphaproteobacterial origin. The taxonomic affiliation of the remaining contigs could not be ascertained. In the crenarchaeal contigs, the majority of the predicted genes had highest similarities to *N. maritimus* and to a lesser extent, to *Cenarchaeum symbiosium* or to uncultured crenarchaotes. All these contigs were low GC%, with the mean GC% of the genes ∼39%, fitting well with the known low GC% of *N. maritimus* genome (34%), but not with *C. symbiosum* (57%). The assembly of these contigs directly from the metagenomic data alone provides convincing evidence that freshwater crenarchaeotes are indeed abundant organisms in the sample and may not be very diverse (compared to others that might be abundant but do not assemble). Remarkably, one of the contigs ascribed to crenarchaea was syntenic to the genomic region in the *N. maritimus* genome containing the important genes for ammonia-oxidation (amoA, amoB and amoC genes) (Figure 6). A search for ammonium monoxygenases (all subunits), using several representative archaeal proteins always retrieved better hits (% similarity ranging from 85% to 98%) than the bacterial homologs that yielded lower similarity levels (% similarity ranging from 45% to 82%), indicating that ammonia-oxidation in this microbial community is a function primarily performed by archaea. Other approaches have shown previously that in marine, freshwater and soil environments archaeal oxidation of ammonia to nitrite has been shown to be dominant over bacterial contribution [40], [43], [45].

An unexpected result from the taxonomic analysis was the presence of a close relative of *Acinetobacter baumannii*, a gammaproteobacterium often isolated from nosocomial infections. The genome of a human isolate, strain ATCC 17978, recruited a high number of reads (2.38% of binned reads) at very high % identity levels (Table 1 and Figure S3). Acinetobacters are routinely isolated from soil and water samples [46], [47]. However, they are not generally detected using PCR-based 16S rRNA gene surveys of freshwater [6], [7] suggesting they are usually present only at low abundance compared to typical freshwater organisms. Thus, the presence of an *Acinetobacter* species sharing so much genetic content with a previously sequenced human isolate in our sample might be the result of contamination during sample manipulation. However, closer inspection of the metagenomic reads affiliating with *Acinetobacter* 16S rRNA genes (72% of gammaproteobacteria-affiliated 16S rRNA reads) showed that nearly half the reads had the highest similarity to several other non-*baumanii Acinetobacter* species making it difficult to definitively conclude the identity of the microbes recruiting the reads. Besides, 16S rRNA gene PCR amplification in a total of four independent samples taken from the river mainstem, at this and other locations revealed large numbers of 16S rRNA that could be assigned to the genus *Acinetobacter*, but not the species *A. baumannii*. (data not shown). In any case, the natural habitat (reservoir) of nosocomial *A. baumannii* has been elusive and there has been recent evidence pointing towards a freshwater origin, e.g. it has been proven that freshwater amoeba can aid the survival of *A. baumannii* by inclusion in cysts [48]. Therefore, the presence in river waters of *A. baumanii* genotypes that might be eventually involved in nosocomial outbreaks is not out of the question. In fact, the ‘outbreak’ of a similarly unlikely nosocomial pathogen has been observed in a pristine South American lagoon system [49].

The genome of the marine, alpha-proteobacterial SAR11 strain *Candidatus* Pelagibacter ubique HTCC1062 also recruited a substantial number of reads from the Amazon dataset (Table 1 and Figure S3) and the Lake Gatun metagenomic dataset (Table S3). The SAR11 lineage is the most dominant bacterioplankton in the oceanic waters and the existence of a freshwater sister group (LD12 or alfV-A) has been known for some time [6], [7]. Genomic sequence information for LD12 is scarce, apart from 16S rRNA gene sequences. However, phylogenetic studies have indicated that there are distinct differences between the freshwater and the marine groups [5]. Both appear to have adapted independently to their niches with almost no mixing despite enormous population sizes. Although the *Candidatus* Pelagibacter ubique HTCC1062 genome recruited many reads from both the Amazon and Lake Gatun datasets, the Alphaproteobacteria in these systems are clearly distinct from the marine SAR11 lineage, sharing 64% mean protein sequence identity with the Pelagibacter genome. Notably, nearly one-third of rhodopsins found in our dataset (41 out of 125) were binned as alphaproteobacterial, indicating that this freshwater clade, like the marine SAR11, might also use rhodopsins for photoheterotrophy.

Seven contigs contained several genes most similar to *Candidatus* Pelagibacter species, supporting the abundant presence of the freshwater group LD12 in the freshwater sample. These contigs were also low GC% (mean GC% was 31%), fitting well with the low GC% of known *Candidatus* Pelagibacter genomes (∼30%). It has been hypothesized that low GC is an adaption by the marine SAR11 clade to counter the low availability of nitrogen in the oligotrophic ocean waters [50]. However, the GC content of the Amazon dataset reads with best hits to this genome was also very low (∼32%), suggesting that the LD12 group also has a low GC content. This section of the Amazon river is unlikely to be nitrogen limited (implying that there may be other factors involved in determining GC content than nitrogen availability alone).

### Signatures of heterotrophy

To explore functional differences between flowing freshwater and marine microbial communities, we compared the Amazon sample to selected, representative marine metagenomic datasets using STAMP [51]. We chose the dataset of the Mediterranean Deep Chlorophyll Maximum (DCM), that was generated in a similar fashion [34], and three warm water sample datasets from a latitude similar to the Amazon from the GOS dataset; GS15, coastal sample from the Caribbean Sea, Off Key West Florida; GS16, a coastal sea sample from and GS26, and open ocean sample 134 miles from the Galapagos Islands. There are fundamental ecosystem-level differences between these marine and the freshwater dataset of the Amazon. For example, the DCM is located in an oligotrophic, phosphate poor environment and has an abundant photosynthetic cyanobacterial population, while phytoplankton production has been found to be limited in the Amazon mainstem, due to turbidity [52]. The DCM is a more stable and a more or less closed system with very low amount of external inputs in the form of organic matter. The three other marine samples are typical oligotrophic sea surface samples as well. The Amazon, however, is a more dynamic, physically mobile system, with continuous terrestrial organic matter inputs.

One of the most striking differences between the Amazon and the marine datasets was a strong signature of microbial heterotrophy in the Amazon (File S1). Marine samples contained more reads from photosystems (I and II) and carotenoid biosynthesis (for primary production), and also of uptake systems for choline and betaine (compatible solutes). The latter are hallmarks of osmotically unbalanced habitats, including seawater. The Amazon sample, on the other hand, was enriched in pathways required for degrading many diverse carbon sources including aromatic compounds, (e.g. phenylacetate, benzoate) and amino acids (e.g. Histidine, Leucine, Isoleucine, Arginine). Formate hydrogenase, the Entner-Doudoroff pathway for glycolysis, and TCA cycle genes were markedly over-represented in the Amazon as compared to the GOS samples. In addition, polyhydroxybutyrate metabolism genes were also more abundant indicating extensive use of this important carbon storage material by the freshwater microbiota. These results are consistent with the Amazon being a net heterotrophic ecosystem. Indeed, the bulk of the organic matter in the river originates from the surrounding forest [53] and others have measured high rates of microbial respiration, even in the absence of high primary production [54].

Closer inspection of the pathways involved in carbon metabolism revealed that microbes in the Amazon are likely processing organic matter fixed by terrestrial plants, providing further evidence for allochthony (File S1). For example, phenylacetate and phenylpropanoids are aromatic secondary metabolites produced by plants [55]. Some of the latter are precursors for the formation of lignin, an important structural polymer in plant cell walls. Lignin is a highly refractory compound and resistant to degradation, but can be degraded by basidiomycete fungi (e.g. white rot) and bacteria. Degradation leads to the formation of ferulic, vanillic and protocatechiuc acids, all phenylpropanoids [56]. These can be used as a substrate by several bacteria as the sole source of carbon [57]. Several key proteins involved in the uptake (a vanillate transporter vanK), and subsequent conversion of ferulic acid to 3-carbon metabolites (Feruloyl-CoA synthetase, phenylpropanoid dioxygenase) were identified as abundant in the Amazon sample. Enzymes in a related pathway, also leading to the transport and production of 3-carbon metabolites from benzoic acid, were also identified. The genes conferring ability to assimilate alkanesulfonates were also overrepresented in the Amazon dataset. Soil is a major reservoir for alkanesulfonates derived originally from plant material [58].

Although it is tempting to conclude that the above characteristics of Amazon microbes can be attributed to the freshwater habitat more generally, we found many of the carbon-processing features were unique even in comparison to Lake Gatun. The Amazon sequences were also comparatively more enriched in genes for aromatic compound degradation, amino acid degradation, polyhydroxybutyrate metabolism, and alkanesulfonate assimilation. This indicates that these represent strategies utilized by the microbial community of the Amazon specifically. However, among the important pathways enriched in the Amazon sample relative to the marine samples, that were also found in Lake Gatun were genes in the Entner-Duodoroff pathway, archaeal Embden-Meyerhoff Pathway and gluconeogenesis, and glycerate metabolism (File S1). This implies fundamental differences in carbon metabolism pathways between freshwater and marine environments. We also did not find any evidence of differences in light-dependent metabolic genes (e.g. photolyases, rhodopsins) except for photosystem II genes which were underrepresented in comparison to Lake Gatun.

We also found genes involved in resistance to cobalt-zinc and cadmium overrepresented in the Amazon alone, compared to all these other datasets, and the majority of these genes could be attributed to betaproteobacteria, that are much more abundant in the Amazon (23% of classifiable reads) than in Lake Gatun (9% of classifiable reads).

To further infer unique functional features of the Amazon river as compared to Lake Gatun, we annotated all the PFAM domains found in the predicted proteins of the two datasets, and identified domains that were more frequent in one dataset versus the other. Among the domains that were markedly more frequent in the Amazon were a number of phage-protein related domains (e.g. coat proteins, terminases, portal proteins) (Table S4 and Table S5), indicating the presence of some Amazon-dataset specific phages. One particularly interesting feature was the overrepresentation of the Tannase domain in the Amazon dataset. This domain is found in proteins involved in degradation of aromatic compounds (e.g. tannins) [59], indicating that much this specific activity is much more common in the Amazon. This is another piece of evidence for the importance of terrestrial organic matter in Amazon carbon cycle. Analysis of the taxonomic distribution of the metagenomic reads revealed this function to be broadly distributed amongst Acidobacteria and Proteobacteria (alpha, beta and gamma) to nearly equal amounts.

However, the most frequently found protein domain in the Amazon dataset, and which was also overrepresented compared to Lake Gatun, was the Bug domain (Table S4), which is found in proteins that are extracytoplasmic tripartite tricarboxylic acid receptors [60]. The substrate binding specificity of these receptors is largely uncharacterized, but citrate, glutamate, and aspartate have been co-crystallized with Bug proteins [61]. This domain was found mainly in betaproteobacterial reads (83% of all reads with the Bug domain), which is consistent with previous studies searching for Bug genes in finished genomes [60]. Their abundance in the dataset indicates that tricarboxylic acids may be a common source of carbon in the flowing river or may simply reflect the abundance of betaproteobacteria in the Amazon (23% of all reads) as compared to Lake Gatun (9% of all reads).

### Concluding Remarks

Much less is known about freshwater bacteria than marine ones, or those that impact human health directly (e.g. pathogenic bacteria). This is evidenced by the paucity of genomic sequences available for freshwater bacteria, for instance, *Polynucleobacter necessarius* is the only cosmopolitan freshwater bacteria that has one strain genome sequenced. This can also be attributed to the hurdles in culturing, which has been the main starting point of nearly all sequenced bacterial genomes. Even metagenomic studies, which provide a culture-free alternative, have been heavily biased towards marine systems. Freshwaters play a previously underestimated but surprisingly important role in the oxidation, storage, and release of terrestrial carbon, thereby affecting global carbon budgets [2], [3], [4]. Our results confirm the importance of heterotrophic metabolism in this large river. Most of the information that has been gathered about the freshwater microbes has been through 16S rRNA gene cloning studies and limited fosmid end sequencing [30], but our results should encourage additional efforts to better characterize the freshwater metagenome in both rivers and lakes, particularly with respect to carbon metabolism.

Importantly, we confirm by a metagenomic approach that actinobacteria are primary members of freshwater ecosystems. The GC content of these relatively unknown actinobacteria does not conform to the generally held views that free living actinobacteria have high GC content [36]. Also, the results indicate that the genomic sequence fragments that we have recovered from these actinobacteria do not correspond to any particular actinobacterial species, but to a multitude, indicating that they are quite divergent from all known actinobacteria.

Apart from actinobacteria, the genomic fragments of the freshwater sister group of *Candidatus* Pelagibacter (LD12) was also found to be quite abundant and distinct from the marine species. Another surprising finding was that maximum number of reads could be assigned not to a bacterial genome, but to the genome of *N. maritimus*, a crenarchaeon. These reads likely belong to a freshwater relative of this marine microbe. Moreover, it appears that crenarchaea in general seem to be much more abundant in freshwaters than in marine systems. These observations taken together indicate the near non existence of closely related genome sequences of microbes belonging to this dataset, and point to the high degree of uniqueness of this sample. This is an even more pressing reason to conduct further meta-'omics based work in these previously neglected ecosystems.

## Materials and Methods

### Sample collection and processing

The sample was collected in accordance with the Brazilian law (IN n° 154/2007 IBAMA, Brazilian Institute of Environment and Renewable Natural Resources). The sample was collected from a depth of 8 m from the Solimões-Amazon River (03°56′11.08″S, 63°10′14.45″W) with a 10 liter Niskin bottle on 17 September, 2008, at 2:00 pm. River depth and width at this location were 15 m and 2100 m respectively, and the sample was taken 600 m away from the shore. Some physico-chemical parameters are shown in Table S1.

Four liters of water were filtered in 3 independent Sterivex filters. Each sample was sequentially filtered through a AP20 glass fiber filter (142 mm of diameter); 5 µm pore size polycarbonate filter (142 mm of diameter) and 0.22 µm-pore size Sterivex GV filters (Durapore, Millipore) using a peristaltic pump. Sterivex filters (retaining the 0.2–5 µm-diameter planktonic cells) were filled up with lysis buffer (40 mM EDTA, 50 mM Tris/HCl, 0.75 M sucrose) and stored at −20°C until DNA extraction. The solution was harvested from the filters and DNA was extracted using the Metagenomic DNA from Environmental Water Samples Kit (EPICENTRE, WI, USA). DNA integrity was checked by agarose gel electrophoresis and quantified spectrophotometrically in a NanoDrop ND 1000 instrument (Thermo Scientific, DE, USA). Five micrograms was used for sequencing in the 454 Sequencing GS FLX Titanium platform (Laboratório Nacional de Computação Científica, Petrópolis, Rio de Janeiro, Brazil).

### Annotation and assembly

The raw metagenomic data were initially processed using CLC Genomics Workbench 3.5, where sequences were quality trimmed. Sequences less than 60 bp were discarded. Assembly was performed using the CLC Genomics Workbench Assembler (>95% identity and >50% of read length). Gene prediction was performed on the assembled sequences using the program MGA (Noguchi *et al.*, 2006). Protein sequences were annotated by using the best blast hit against the NCBI NR database. The entire unassembled dataset was also annotated using the MG-RAST server [62].

*Community structure using all reads*. For taxonomy, the dataset was compared using BLAST [63] to a combined database containing the NCBI- NT database and whole genome shotgun assembly data for 1000 draft microbial genomes from NCBI (http://www.ncbi.nlm.nih.gov/lproks.cgi). The data were analyzed using MEGAN [64]. The taxonomic analysis was also performed using the MG-RAST server using a cutoff of minimum alignment length ∼50 and an evalue cutoff of 1e-5.

### Community structure using rRNA

Ribosomal rRNA gene sequences in the metagenomic data were identified by comparison against the RDP [65] using BLASTN. Only alignments longer than 100 bp were considered. Sequences were considered identified to the genus level if they had a %identity >95% with a well identified existing sequence that was not annotated as “uncultured” or “unidentified”. The best named hit to the metagenomic rRNA sequence was considered to assign the sequence to a high-level taxonomic group. Otherwise only higher taxonomic levels were used. Sequences that matched only those annotated as “unidentified” or “uncultured” and those with <90% identity to the best matching hit were considered unidentified.

### Recruitment plots

Fragment recruitment of the Amazon dataset was performed against all complete and draft microbial genomes using BLASTN. The criteria for counting a hit were minimum %identity of 95% and minimum alignment of 50 bp. Data was plotted using R (http://cran.r-project.org). For selected genomes, recruitment was also performed using TBLASTX (evalue cutoff 1e-5 and minimum alignment length 50).

### Comparison with GOS dataset

The Amazon data were compared against the entire Global Ocean Sampling (GOS) expedition data [28] using BLASTN, and a hit was counted using the criteria of minimum 95% identity and alignment length of at least 50 bases.

### Clustering with selected metagenomic datasets

All vs all comparison was performed using BLASTN with all selected datasets. Only blast hits with >70% identity and >100 bp length were considered. The Jaccard distance *D* between two datasets (*A* and *B*) can be computed as follows
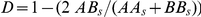
where *AB_s_* is the total bit score of all the common hits between datasets *A* and *B*, *AA_s_* and *BB_s_* are the total bit scores of the comparisons of the datasets *A* and *B* to themselves. The tree was built using the neighbor program in the PHYLIP package.

### Functional profile comparison

Comparison of functional profiles was performed using STAMP [51]. The minimum %identity was 70% and the minimum alignment length was ∼100. Statistical significance of the differences between samples was assessed by the Two-sided Fisher's Exact test and Storey's FDR method was used for multiple test correction The most important metabolic categories were selected by filtering by q-value (0.05), and using only those categories that had at least 100 sequences and more than 2-fold ratio between the proportions.

### Pfam Domain analysis

Orf prediction in the metagenomic reads was performed using FragGeneScan [66] and HMMER3 package was used to identify Pfam domains in translated protein sequences >60 aa in length. In the comparisons to detect more frequent protein domains in dataset X versus dataset Y, the abundance ratio is computed as % of domain in dataset X/% of domain in dataset Y.

### Accession numbers

Sequence data have been deposited in the INSDC Sequence Read Archive under the accession SRP005263.2.

## Supporting Information

Figure S1 **Location where the sample was taken.** The red arrow in the magnified view (right panel) marks the location of the site.
(TIF)

Figure S2 **Comparison of Amazon dataset to the entire GOS dataset.** Comparison done using BLASTN. Minimum criteria for counting a hit were %identity > = 90%, and minimum alignment length of 50 bases. Data shown are hits to each dataset (using Amazon metagenome as query) normalized by the total number of sequences in each GOS sample. Only the top 10 samples are represented here. Shown above each bar are the Latitude, Longitude, Sample Depth, Chlorophyll Content, Salinity, Temperature and Date of Collection. (NA: data not available)
(TIF)

Figure S3 **Recruitment of Amazon metagenome reads by microbial genomes (** ***Nitrosopumilus maritimus*** **,** ***Polynucleobacter necessarius*** **QLW-P1DMWA,** ***Candidatus*** **Pelagibacter ubique HTCC1062 and** ***Acinetobacter baumannii*** **ATCC 17978).** The vertical axis represents the %identity of the metagenomic read to the genome. The comparison was made using TBLASTX.
(TIF)

Figure S4 **Phylogenetic profile of the Low GC (<50% GC) and the High GC (>50% GC) reads of the amazon metagenome.** Total low GC reads (including unclassified) = 594257, Total high GC reads (including unclassified) = 559245
(TIF)

Figure S5 **Archaeal reads in diverse metagenomes.** A) Archaeal vs Bacterial Reads across several metagenomic datasets (shown as a % of all reads with a hit at evalue <1e-5 and alignment length 50). B) Comparison of Archaeal taxonomic groups across several metagenomic datasets (shown as a % of all archaeal reads). Nanoarchaeota not shown as they comprised less than 1% reads in all datasets)
(TIF)

Table S1 **Sample parameters.**
(DOCX)

Table S2 **Phylogenetic Profile of reads common between Amazon and Lake Gatun.**
(DOCX)

Table S3 **Phylogenetic Profile of Lake Gatun using the MG-RAST Server.**
(DOCX)

Table S4 **Protein domains overrepresented in the Amazon dataset versus Lake Gatun dataset.**
(DOCX)

Table S5 **Protein domains overrepresented in the Lake Gatun dataset versus Lake Gatun dataset.**
(DOCX)

File S1 **The file contains the results of the STAMP comparison of the Amazon dataset versus the other datasets (Lake Gatun, Deep Chlorophyll Maximum and 3 GOS samples GS15, GS16 and GS26).**
(XLSX)
