# Correction: Metagenomics of the Water Column in the Pristine Upper Course of the Amazon River

**DOI:** 10.1371/journal.pone.0097393

**Published:** 2014-05-06

**Authors:** The PLOS ONE Staff

The second author’s name is spelled incorrectly. The correct name is: Francisco Rodriguez-Valera.
