# Supplementary figures and images for: Metagenomics of the Water Column in the Pristine Upper Course of the Amazon River
Source: PLoS One. 2011 Aug 19;6(8):e23785. doi: 10.1371/journal.pone.0023785 (PMC3158796; doi:10.1371/journal.pone.0023785)

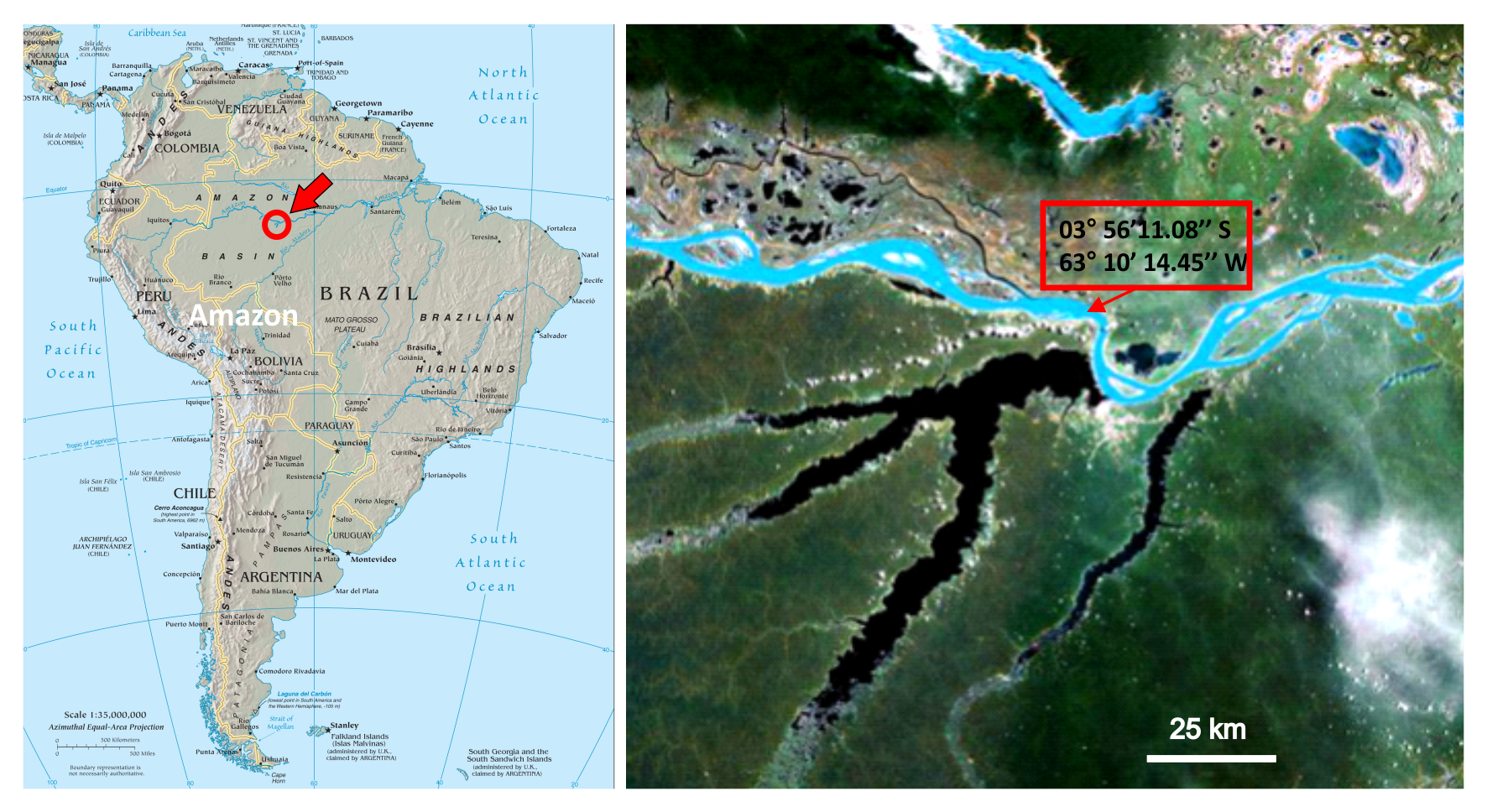

Supplement: Figure S1 — Location where the sample was taken. The red arrow in the magnified view (right panel) marks the location of the site. (TIF) [file pone.0023785.s001.tif]

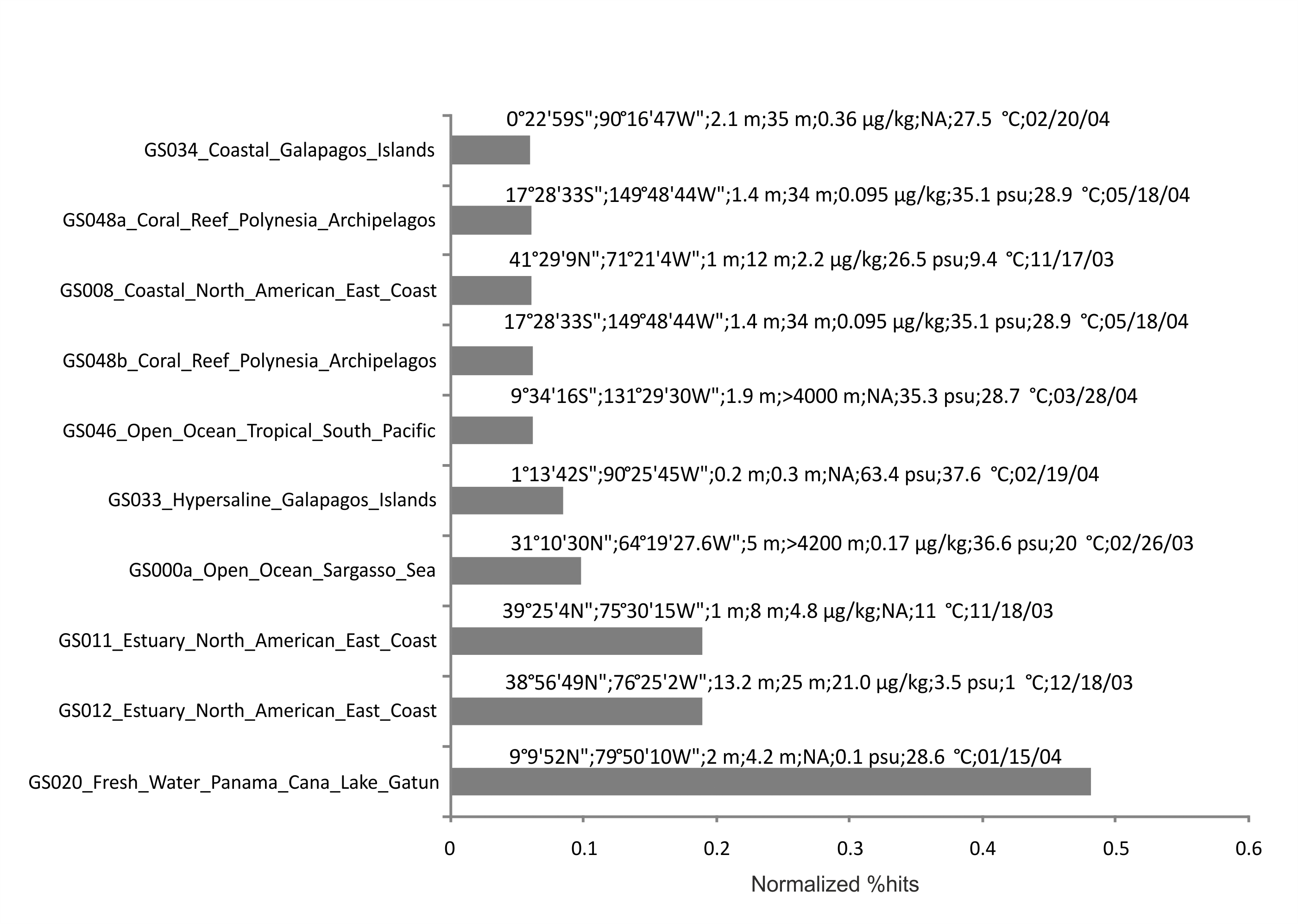

Supplement: Figure S2 — Comparison of Amazon dataset to the entire GOS dataset. Comparison done using BLASTN. Minimum criteria for counting a hit were %identity > = 90%, and minimum alignment length of 50 bases. Data shown are hits to each dataset (using Amazon metagenome as query) normalized by the total number of sequences in each GOS sample. Only the top 10 samples are represented here. Shown above each bar are the Latitude, Longitude, Sample Depth, Chlorophyll Content, Salinity, Temperature and Date of Collection. (NA: data not available) (TIF) [file pone.0023785.s002.tif]

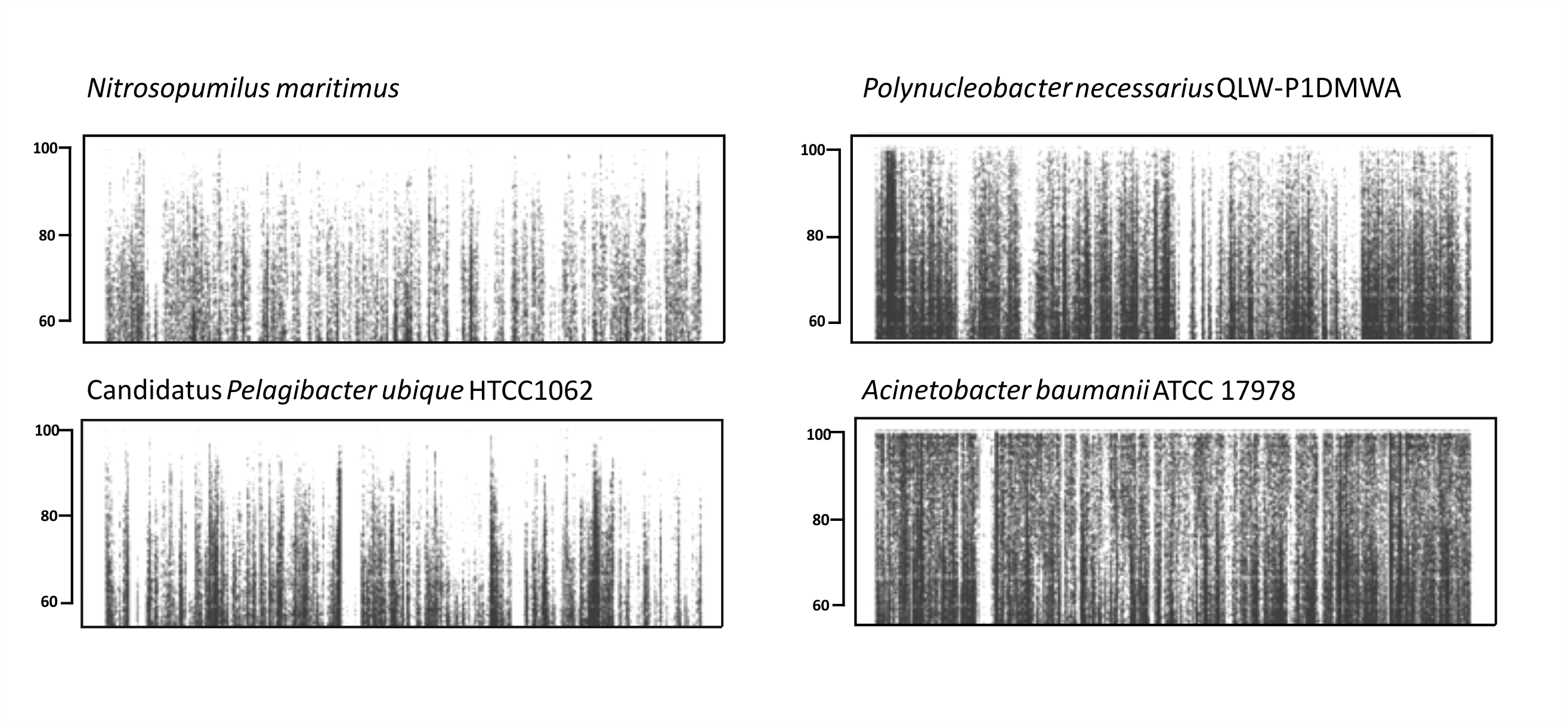

Supplement: Figure S3 — Recruitment of Amazon metagenome reads by microbial genomes ( Nitrosopumilus maritimus , Polynucleobacter necessarius QLW-P1DMWA, Candidatus Pelagibacter ubique HTCC1062 and Acinetobacter baumannii ATCC 17978). The vertical axis represents the %identity of the metagenomic read to the genome. The comparison was made using TBLASTX. (TIF) [file pone.0023785.s003.tif]

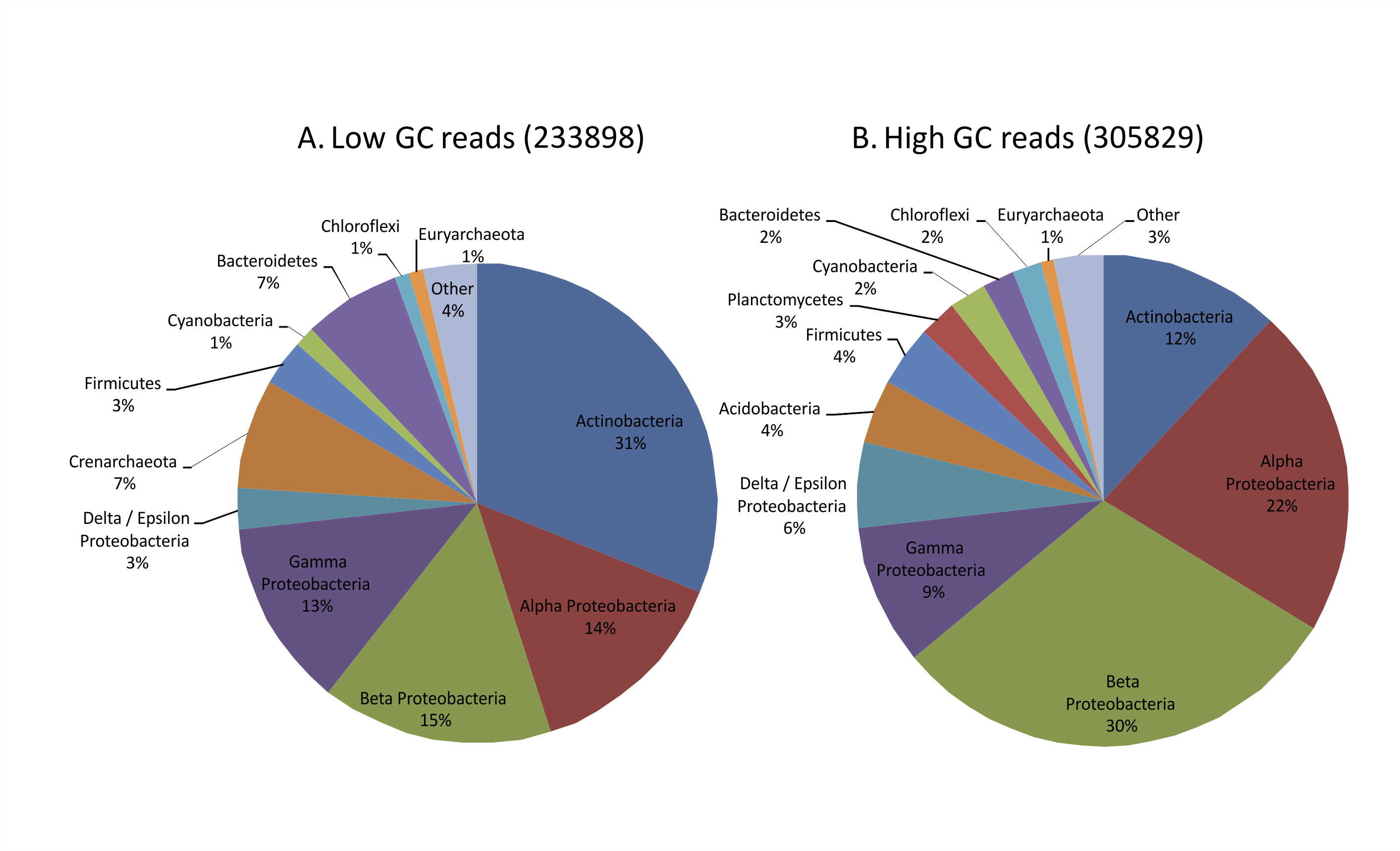

Supplement: Figure S4 — Phylogenetic profile of the Low GC (<50% GC) and the High GC (>50% GC) reads of the amazon metagenome. Total low GC reads (including unclassified) = 594257, Total high GC reads (including unclassified) = 559245 (TIF) [file pone.0023785.s004.tif]

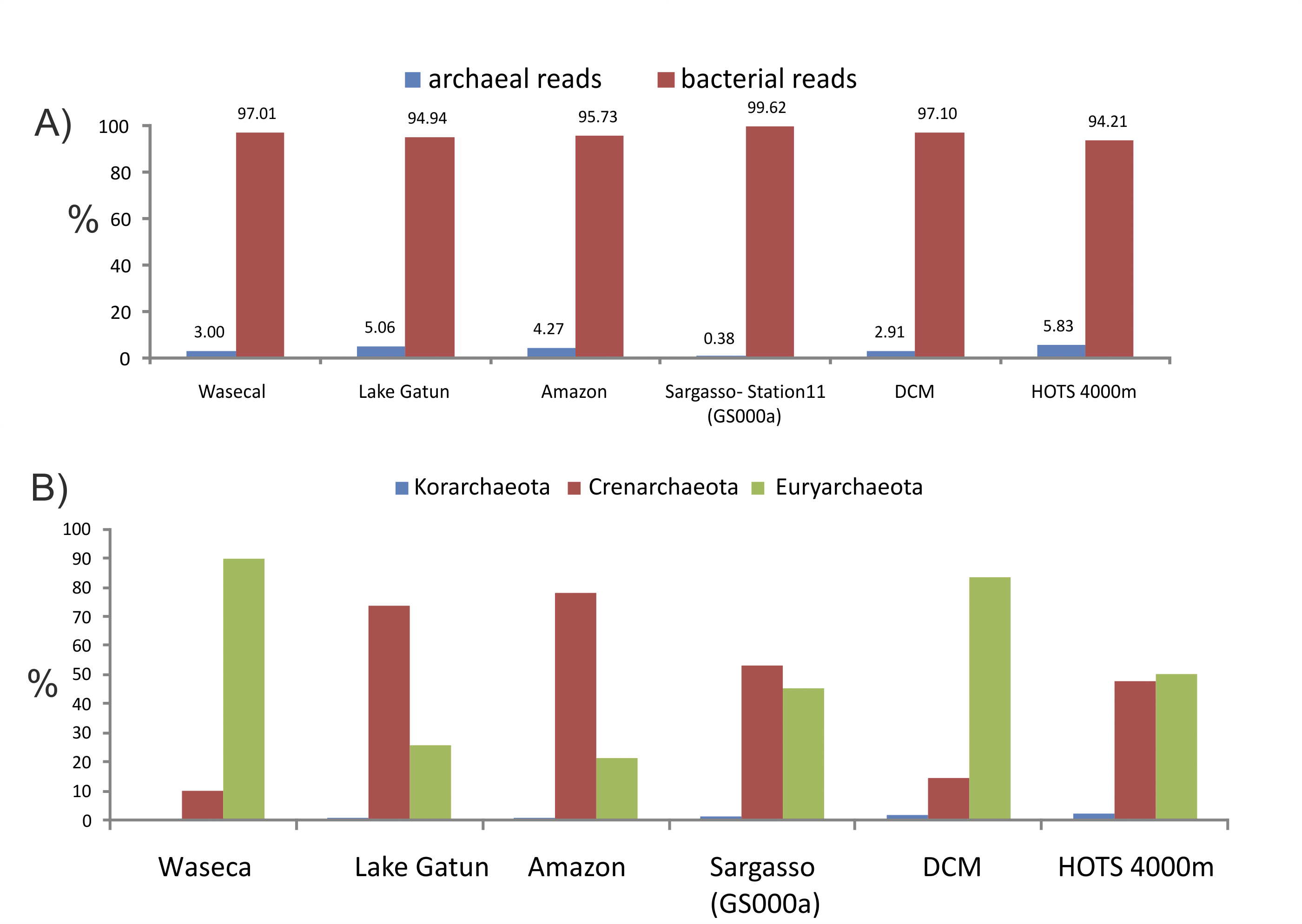

Supplement: Figure S5 — Archaeal reads in diverse metagenomes. A) Archaeal vs Bacterial Reads across several metagenomic datasets (shown as a % of all reads with a hit at evalue <1e-5 and alignment length 50). B) Comparison of Archaeal taxonomic groups across several metagenomic datasets (shown as a % of all archaeal reads). Nanoarchaeota not shown as they comprised less than 1% reads in all datasets) (TIF) [file pone.0023785.s005.tif]
