# Supplementary material for: Metagenomics of the Water Column in the Pristine Upper Course of the Amazon River
Source: PLoS One. 2011 Aug 19;6(8):e23785. doi: 10.1371/journal.pone.0023785 (PMC3158796; doi:10.1371/journal.pone.0023785)
Supplement: Table S1 — Sample parameters. (DOCX) [file pone.0023785.s006.docx]

**Table S1:** Sample parameters

| Sample Collection Depth (m) | 8 |
| --- | --- |
| Total Depth (m) | 15 |
| River Width(m) | 2100 |
| Temperature (⁰C) | 29.8 |
| pH | 7.09 |
| Oxygen (mg L^-1^) | 5.05 |
| Turbidity (NTU) | 18.20 |
| Suspended material (mg L^-1^) | 45.67 |
| Biological Oxygen Demand (mg L^-1^) | -2.90 |
| Transparency (m) | 0.2 |
| Conductivity (µS cm^-1^) | 109.7 |
| Latitude and Longitude | 03°56'11.08'' S  63°10'14.45'' W |
| Collection date | 17 September 2008 |
| Collection time | 2:00 pm |
