# Supplementary material for: Metagenomics of the Water Column in the Pristine Upper Course of the Amazon River
Source: PLoS One. 2011 Aug 19;6(8):e23785. doi: 10.1371/journal.pone.0023785 (PMC3158796; doi:10.1371/journal.pone.0023785)
Supplement: Table S2 — Phylogenetic Profile of reads common between Amazon and Lake Gatun. (DOCX) [file pone.0023785.s007.docx]

**Table S2:** Phylogenetic Profile of reads common between Amazon and Lake Gatun

| **AMAZON READS COMMON WITH LAKE GATUN** |  | **LAKE GATUN READS COMMON WITH AMAZON** |  |
| --- | --- | --- | --- |
| NAME | # HITS | NAME | # HITS |
| Nitrosopumilus maritimus SCM1 | 11676 | Candidatus Pelagibacter ubique HTCC1062 | 12033 |
| Candidatus Pelagibacter ubique HTCC1062 | 11613 | Acidothermus cellulolyticus 11B | 8584 |
| Acidothermus cellulolyticus 11B | 3712 | Nitrosopumilus maritimus SCM1 | 7191 |
| Streptomyces scabiei str. 87.22 | 2903 | Streptomyces avermitilis MA-4680 | 6696 |
| Janibacter sp. HTCC2649 | 2828 | Streptomyces scabiei str. 87.22 | 6168 |
| Polynucleobacter sp. QLW-P1DMWA-1 | 2806 | Janibacter sp. HTCC2649 | 5891 |
| Methylobacillus flagellatus KT | 2767 | Thermobifida fusca YX | 5885 |
| Thermobifida fusca YX | 2667 | Streptomyces coelicolor A3(2) | 5691 |
| Streptomyces avermitilis MA-4680 | 2612 | Frankia sp. EAN1pec | 4729 |
| Streptomyces coelicolor A3(2) | 2386 | Kineococcus radiotolerans SRS30216 | 4422 |
| Kineococcus radiotolerans SRS30216 | 2142 | Frankia sp. Ccl3 | 3303 |
| Frankia sp. EAN1pec | 2028 | Salinispora arenicola CNS-205 | 3134 |
| Salinispora tropica CNB-440 | 1516 | Salinispora tropica CNB-440 | 2899 |
| Frankia sp. Ccl3 | 1405 | Methylobacillus flagellatus KT | 2551 |
| Salinispora arenicola CNS-205 | 1258 | Solibacter usitatus Ellin6076 | 2465 |
| Nocardia farcinica IFM 10152 | 1071 | Cytophaga hutchinsonii ATCC 33406 | 2439 |
| marine actinobacterium PHSC20C1 | 924 | Polynucleobacter sp. QLW-P1DMWA-1 | 2352 |
| Brevibacterium linens BL2 | 874 | Nocardia farcinica IFM 10152 | 2244 |
| Mycobacterium smegmatis str. MC2 155 | 823 | marine actinobacterium PHSC20C1 | 1997 |
| Parvibaculum lavamentivorans DS-1 | 786 | Blastopirellula marina DSM 3645 | 1913 |
| Mycobacterium vanbaaleni vanbaalenii PYR-1 | 741 | Rubrobacter xylanophilus DSM 9941 | 1876 |
| Clavibacter michiganensis subsp. michiganensis NCPPB 382 | 633 | Mycobacterium smegmatis str. MC2 155 | 1693 |
| Propionibacterium acnes KPA171202 | 613 | Mycobacterium vanbaaleni vanbaalenii PYR-1 | 1629 |
| Rubrobacter xylanophilus DSM 9941 | 591 | Acidobacteria bacterium Ellin345 | 1617 |
| Polaromonas sp. JS666 | 578 | Brevibacterium linens BL2 | 1498 |
| Magnetospirillum magnetotacticum | 547 | Parvibaculum lavamentivorans DS-1 | 1474 |
| Mycobacterium sp. MCS | 505 | Polaromonas sp. JS666 | 1440 |
| Leifsonia xyli subsp. xyli str. CTCB07 | 490 | Sorangium cellulosum So ce 56 | 1343 |
| Solibacter usitatus Ellin6076 | 477 | Roseiflexus sp. RS-1 | 1315 |
| Mycobacterium marinum M | 422 | Herpetosiphon aurantiacus ATCC 23779 | 1270 |
