# Supplementary material for: Metagenomics of the Water Column in the Pristine Upper Course of the Amazon River
Source: PLoS One. 2011 Aug 19;6(8):e23785. doi: 10.1371/journal.pone.0023785 (PMC3158796; doi:10.1371/journal.pone.0023785)
Supplement: Table S3 — Phylogenetic Profile of Lake Gatun using the MG-RAST Server. (DOCX) [file pone.0023785.s008.docx]

**Supplementary Table 3** : Phylogenetic profile of Lake Gatun dataset using the MG-RAST server.

| **Domain** |  | **Organism Name** | **# Hits** |
| --- | --- | --- | --- |
| Bacteria | Proteobacteria | Candidatus Pelagibacter ubique HTCC1062 | 12033 |
| Viruses | dsDNA viruses, no RNA stage | Cyanophage P-SSM2. | 8620 |
| Bacteria | Actinobacteria | Acidothermus cellulolyticus 11B | 8584 |
| Archaea | Crenarchaeota | Nitrosopumilus maritimus SCM1 | 7148 |
| Bacteria | Actinobacteria | Streptomyces avermitilis MA-4680 | 6696 |
| Bacteria | Actinobacteria | Streptomyces scabiei str. 87.22 | 6168 |
| Bacteria | Actinobacteria | Janibacter sp. HTCC2649 | 5891 |
| Bacteria | Actinobacteria | Thermobifida fusca YX | 5885 |
| Bacteria | Actinobacteria | Streptomyces coelicolor A3(2) | 5691 |
| Bacteria | Actinobacteria | Frankia sp. EAN1pec | 4729 |
| Bacteria | Actinobacteria | Kineococcus radiotolerans SRS30216 | 4422 |
| Bacteria | Actinobacteria | Frankia sp. Ccl3 | 3303 |
| Bacteria | Actinobacteria | Salinispora arenicola CNS-205 | 3134 |
| Bacteria | Actinobacteria | Salinispora tropica CNB-440 | 2899 |
| Viruses | dsDNA viruses, no RNA stage | Bacteriophage S-PM2. | 2871 |
| Bacteria | Proteobacteria | Methylobacillus flagellatus KT | 2551 |
| Bacteria | Fibrobacteres/Acidobacteria group | Solibacter usitatus Ellin6076 | 2465 |
| Bacteria | Bacteroidetes/Chlorobi group | Cytophaga hutchinsonii ATCC 33406 | 2439 |
| Bacteria | Proteobacteria | Polynucleobacter sp. QLW-P1DMWA-1 | 2352 |
| Bacteria | Actinobacteria | Nocardia farcinica IFM 10152 | 2244 |
