# Supplementary material for: Metagenomics of the Water Column in the Pristine Upper Course of the Amazon River
Source: PLoS One. 2011 Aug 19;6(8):e23785. doi: 10.1371/journal.pone.0023785 (PMC3158796; doi:10.1371/journal.pone.0023785)
Supplement: Table S4 — Protein domains overrepresented in the Amazon dataset versus Lake Gatun dataset. (DOCX) [file pone.0023785.s009.docx]

**Supplementary Table 4**: Top 25 protein domains overrepresented in Amazon versus Lake Gatun. Column 1 shows the name of the protein domain, columns 2,3 show the number of hits to domain in each dataset. %Ratio= % of domain in Amazon / % of domain in Lake Gatun.

| NAMES | # AMAZON | # GATUN | % Ratio | Comment |
| --- | --- | --- | --- | --- |
| ERF | 203 | 3 | 50.80 | DNA Binding/Recombination |
| **P22_CoatProtein** | 225 | 5 | 33.79 | **Phage** |
| **Phage_portal_2** | 235 | 6 | 29.41 | **Phage** |
| ***Tannase*** | 109 | 3 | 27.28 | ***Degradation of aromatics*** |
| RecT | 148 | 6 | 18.52 | DNA Binding/Recombination |
| **Terminase_3** | 208 | 12 | 13.01 | **Phage** |
| **Head-tail_con** | 237 | 16 | 11.12 | **Phage** |
| MCPsignal | 190 | 13 | 10.97 | Signalling via Histidine Kinases |
| **Terminase_GpA** | 231 | 18 | 9.64 | **Phage** |
| PQQ_N | 175 | 15 | 8.76 | Coenzyme pyrrolo-quinoline quinone |
| Laminin_G_1 | 100 | 9 | 8.34 | Extracellular Domain (usually Metazoan) |
| VRR_NUC | 115 | 11 | 7.85 | Type III restriction modification enzymes associated domain |
| DUF3458 | 91 | 9 | 7.59 | Domain of unknown function |
| HSP90 | 202 | 20 | 7.58 | Heat Shock Protein |
| HDOD | 147 | 16 | 6.90 | Phosphohydrolases in nucleic acid metabolism |
| **Bug** | **5644** | 645 | 6.57 | **Extracytoplasmic tricarboxylate-binding receptor** |
| CheW | 113 | 13 | 6.53 | Signalling via Histidine Kinases |
| HemolysinCabind | 333 | 41 | 6.10 | Hemolysin-type calcium-binding repeat |
| Transposase_mut | 137 | 18 | 5.71 | Transposase |
| FHIPEP | 185 | 25 | 5.56 | Type III Secretion Apparatus |
| DUF847 | 111 | 15 | 5.56 | Lysozyme related enzyme |
| **ACR_tran** | **2567** | 363 | 5.31 | **Integral Membrane proteins/Drug Efflux** |
| **Terminase_1** | 284 | 43 | 4.96 | **Phage** |
| DUF404 | 99 | 15 | 4.96 | Domain of unknown function |
| RusA | 131 | 20 | 4.92 | Endonuclease |
