# Supplementary material for: Metagenomics of the Water Column in the Pristine Upper Course of the Amazon River
Source: PLoS One. 2011 Aug 19;6(8):e23785. doi: 10.1371/journal.pone.0023785 (PMC3158796; doi:10.1371/journal.pone.0023785)
Supplement: Table S5 — Protein domains overrepresented in the Lake Gatun dataset versus Lake Gatun dataset. (DOCX) [file pone.0023785.s010.docx]

**Supplementary Table 5**: Top 25 protein domains overrepresented in Lake Gatun versus Amazon. Column 1 shows the name of the protein domain, columns 2,3 show the number of hits to domain in each dataset. %Ratio= % of domain in Lake Gatun / % of domain in Amazon.

| NAMES | # AMAZON | # GATUN | % Ratio | Comment |
| --- | --- | --- | --- | --- |
| DUF3110 | 5 | 99 | 26.37 | Domain of unknown function |
| PduV-EutP | 29 | 124 | 5.70 | Ethanolamine Utilization |
| Glyco_transf_11 | 45 | 189 | 5.59 | Glycosyltransferase |
| Glyco_transf_25 | 52 | 182 | 4.66 | Glycosyltransferase |
| Ras | 43 | 148 | 4.58 | GTPase |
| Actin | 39 | 126 | 4.30 | Structural Polymer |
| Sulfotransfer_2 | 27 | 82 | 4.05 | Sulfotransferase |
| TnsA_N | 35 | 98 | 3.73 | Transposase |
| ATP-grasp_3 | 53 | 147 | 3.69 | Ligation of carboxylate-containing molecule to thiol group using ATP hydrolysis |
| 2OG-FeII_Oxy | 74 | 200 | 3.60 | Oxygenase |
| Glyco_hydro_2 | 30 | 80 | 3.55 | Glycosyl Hydrolase |
| DUF2596 | 33 | 80 | 3.23 | Domain of unknown function |
| Arf | 32 | 77 | 3.20 | GTPase |
| DnaJ_CXXCXGXG | 72 | 172 | 3.18 | DnaJ Chaperone |
| YhjQ | 68 | 159 | 3.11 | Probable Bacterial cellulose synthase related |
| MraY_sig1 | 44 | 102 | 3.09 | Peptidoglycan Biosynthesis |
| PcrB | 68 | 155 | 3.04 | Probable Helicase |
| Sulfotransfer_1 | 42 | 95 | 3.01 | Sulfotransferase |
| DUF1731 | 33 | 73 | 2.95 | Domain of unknown function |
| zf-C3HC4 | 32 | 70 | 2.91 | Zinc finger proteins |
| **Phage_T4_gp19** | 150 | 328 | 2.91 | **Phage** |
| GPW_gp25 | 96 | 209 | 2.90 | Lysozyme related phage protein |
| SNase | 93 | 197 | 2.82 | Nuclease |
| Arg_repressor | 35 | 73 | 2.78 | Arginine Breakdown |
| DUF836 | 35 | 73 | 2.78 | Domain of unknown function |
